# Beyond Accuracy: Perioperative Efficiency and Institutional Cost Implications of CAD/CAM-Guided Versus Conventional Freehand Fibula Free Flap Reconstruction for Mandibular and Maxillary Defects

**DOI:** 10.3390/jcm15051778

**Published:** 2026-02-26

**Authors:** Borja González Moure, Saad Khayat, Diego Fernández Acosta, Ignacio Navarro Cuéllar, Cristina Maza Muela, Ana López López, Manuel Tousidonis Rial, Gema Arenas de Frutos, Carlos Martínez Martínez, Raúl Antúnez-Conde, Stefania Troise, Luigi Angelo Vaira, Giovanni Dell’Aversana Orabona, Santiago Ochandiano, Francisco Javier López de Atalaya, José Ignacio Salmerón, Carlos Navarro Cuéllar

**Affiliations:** 1 Department of Oral and Maxillofacial Surgery, Gregorio Marañon University Hospital, 28007 Madrid, Spain; borjagonzalezuam@gmail.com (B.G.M.); diegofernandezacosta77@gmail.com (D.F.A.); nnavcu@hotmail.com (I.N.C.); crismazamuela@gmail.com (C.M.M.); anitalopez@gmail.com (A.L.L.); gema.arenas@gmail.com (G.A.d.F.); carloshugomar@gmail.com (C.M.M.); antunezconde_92@hotmail.com (R.A.-C.); sochandiano@hotmail.com (S.O.); atalaya-la@hotmail.com (F.J.L.d.A.); jisalmeron@telefonica.net (J.I.S.); 2 Gregorio Marañón Research Institute, 28007 Madrid, Spain; 3 Department of Surgery, Universidad Complutense de Madrid, 28040 Madrid, Spain; 4 Maxillofacial Surgery Unit, Department of Neurosciences, Reproductive and Odontostomatological Sciences, University of Naples Federico II, 80131 Naples, Italy; stefania.troise@unina.it (S.T.); giovanni.dellaversanaorabona@unina.it (G.D.O.); 5 Maxillofacial Surgery Operative Unit, Department of Medicine, Surgery and Pharmacy, University of Sassari, 07100 Sassari, Italy; lavaira@uniss.it

**Keywords:** fibula free flap, CAD/CAM, mandibular reconstruction, virtual surgical planning, perioperative efficiency, cost analysis, value-based surgery

## Abstract

**Background:** Computer-aided design and manufacturing (CAD/CAM) technology has been increasingly adopted for mandibular and maxillary reconstruction using fibula free flaps. However, its clinical and economic advantages over the conventional freehand technique remain debated. The objective of this study was to compare perioperative outcomes and institutional costs between CAD/CAM-guided and conventional freehand fibula flap reconstructions. **Methods:** An ambispective observational study was conducted including patients who underwent mandibular or maxillary reconstruction with an osteocutaneous free fibula flap between 2017 and 2024. Reconstructions were performed either using CAD/CAM-guided virtual surgical planning or the conventional freehand technique. Demographic data, perioperative variables, postoperative outcomes, oncologic margin status, transfusion requirements, and total institutional costs were analyzed. Univariate comparisons were performed between groups, and multivariate linear regression models were used to assess the independent association of CAD/CAM guidance with operative time and hospital length of stay. **Results:** Fifty-one patients were included (25 CAD/CAM-guided and 26 freehand). CAD/CAM-guided reconstruction was associated with a significantly shorter operative time (542.3 ± 65.8 vs. 604.9 ± 79.5 min; *p* = 0.0036) and a shorter overall hospital stay (19.6 ± 7.2 vs. 30.6 ± 26.2 days; *p* = 0.047) in univariate analysis. The need for perioperative blood transfusion was significantly lower in the CAD/CAM group. No significant differences were observed in ICU stay, flap failure, reintervention rate, or postoperative hemoglobin decrease. Although margin status did not differ significantly between groups, a higher proportion of negative margins was observed in the CAD/CAM cohort. In multivariate analysis adjusting for age and perioperative variables, CAD/CAM guidance remained independently associated with reduced operative time, but not with hospital length of stay. Despite higher upfront planning costs, total per-patient cost was lower in the CAD/CAM group due to improved perioperative efficiency. **Conclusions:** CAD/CAM-guided fibula free flap reconstruction is a safe and effective technique that is associated with reduced operative time and lower transfusion requirements while maintaining comparable oncologic outcomes. When perioperative efficiency gains are achieved, these advantages may offset the higher planning costs, resulting in overall cost savings at the institutional level. CAD/CAM-assisted reconstruction may therefore be particularly advantageous in high-volume oncologic centers and anatomically complex cases.

## 1. Introduction

The osteocutaneous free fibula flap (FFF) has been established as the gold standard for mandibular and maxillary reconstruction of extensive defects since the late 20th century, owing to its reliability and the ability to restore the full mandibular curvature through multiple bone segments. Historically, its application was pioneered by Taylor in 1975 [1] as a pure osseous flap, and later refined by Wei in 1986 [1] through the definition of perforating vessels, enabling the addition of a cutaneous paddle. The technique was consolidated for mandibular reconstruction following the landmark reports by Hidalgo in 1989 [1] and Wei’s first case series in 1994 [2]. The primary goal of reconstruction is to achieve optimal functional and aesthetic outcomes, ensuring satisfactory postoperative quality of life [1].

Traditionally, reconstruction of such a complex three-dimensional structure was performed using the “freehand” technique, in which bone segmentation and flap modeling relied heavily on the surgeon’s intraoperative skill, judgment, and a largely empirical “trial-and-error” process. This dependence may lead to prolonged operative time, increased technical difficulty, and reduced reliability in complex cases [1].

Reducing surgical time is a universal goal for both surgeons and institutions, as prolonged operative duration represents an independent and potentially modifiable risk factor for complications. Specifically, it has been demonstrated that the likelihood of complications increases significantly with longer operative time, doubling when thresholds exceed two hours, with a 14% increase in complication probability for every additional 30 min of surgery [3]. Moreover, the economic cost rises by approximately 30–50 € per minute in the operating room [4], 500–600 € per day in the standard hospitalization, and 1.400 € per day in the intensive care unit (ICU).

With the development of computer-aided design and computer-aided manufacturing (CAD/CAM) technology, mandibular and maxillary reconstruction have evolved substantially [5]. Virtual surgical planning (VSP) allows preoperative simulation of the procedure and transfer of this plan to the operative field through stereolithographic models, cutting guides, and pre-bent or patient-specific reconstruction plates (Patient-Specific Implants, PSI).

Despite the reported advantages, rigorous comparison of these two approaches in terms of key clinical and economic variables remains essential. Therefore, the objective of this study is to compare postoperative clinical outcomes and economic costs between mandibular or maxillary reconstruction using the free fibula flap performed with the conventional freehand technique versus the CAD/CAM-guided and virtually planned technique.

The aim of this study was to compare perioperative outcomes and institutional costs between CAD/CAM-guided and conventional freehand fibula free flap reconstruction of the jaw, including both mandibular and maxillary defects, in order to evaluate whether digital planning translates into measurable clinical efficiency and value while maintaining oncologic safety.

## 2. Materials and Methods

### 2.1. Study Design and Sample

The study was designed as an ambispective observational investigation, combining retrospective data collection with prospective follow-up and prospective data collection. The study period (January 2017–December 2024) reflects the full timeframe of patient inclusion and outcome assessment. Data corresponding to the interval from January 2017 through April 2022 were obtained retrospectively from existing clinical records and institutional databases, involving no direct patient contact or intervention after the Ethical approval.

Ethical approval, granted on 1 May 2022, was obtained prior to the initiation of any prospective data collection, patient follow-up, or analysis involving identifiable information.

The Institutional Review Board specifically authorized retrospective review of previously recorded data and approved continuation of the study prospectively until December 2024. Therefore, all procedures complied with ethical standards, and no study-related activities requiring prior approval were conducted before the approval date.

Patients were allocated to the reconstruction group according to the period of surgery. Between 2017 and 2020, all reconstructions were performed using the conventional freehand technique. From January 2021 onward, CAD/CAM-guided reconstruction based on virtual surgical planning became the standard institutional approach and was applied to all eligible patients. This temporal allocation reflects the progressive institutional adoption of CAD/CAM technology and resulted in a non-randomized historical cohort comparison.

Inclusion criteria were: (1) patients aged 18 years or older; (2) mandibular or maxillary defects requiring reconstruction with an osteocutaneous free fibula flap; and (3) availability of complete perioperative clinical and economic data. Both primary and secondary reconstructions were included.

Exclusion criteria were: (1) patients younger than 18 years; (2) reconstructions performed using free flaps other than the fibula; (3) partial-thickness defects not requiring segmental bony reconstruction; and (4) incomplete clinical or cost-related data.

All patients underwent standardized preoperative evaluation, including panoramic radiography and contrast-enhanced computed tomography of the head and neck for assessment of the defect and recipient vessels, as well as computed tomography angiography of the lower limbs for evaluation of donor-site vasculature. Preoperative anesthetic assessment was performed in all cases according to institutional protocols.

### 2.2. Variables of Comparison

The variables analyzed included demographic characteristics (age, sex, cardiovascular risk factors), indication for surgery (malignant tumor, benign lesion, post-traumatic defect), need for neck dissection or tracheostomy, total operative time (from initial incision to closure), hospital and ICU stay, postoperative laboratory data, need for blood transfusion, complications, histopathology (negative margins, defined as a clearance greater than 5 mm), and total cost per patient.

### 2.3. Cost Analysis

The economic evaluation was performed from an institutional perspective and focused on direct hospital-related costs. Real internal cost estimates were used to calculate the mean total cost per patient in each group. Cost components included operating-room time, intensive care unit (ICU) stay, standard ward hospitalization, perioperative blood transfusions, and reconstruction-related device costs.

Operating-room time was valued per minute, while ICU and ward stays were calculated on a per-day basis according to standardized institutional cost estimates. Blood transfusion costs were calculated per unit transfused. Device-related costs included virtual surgical planning, cutting guides, and patient-specific implants for the CAD/CAM group, and standard reconstruction plates and fixation materials for the conventional freehand group.

The analysis was descriptive in nature, and no inferential statistical testing was performed for cost comparisons. Indirect costs, outpatient care, rehabilitation, and long-term follow-up expenses were not included.

Because patient allocation followed a temporal before–after design, potential changes in institutional cost structures over time may have influenced the economic estimates. Therefore, cost results should be interpreted as indicative of relative institutional resource utilization rather than as definitive cost-effectiveness measures.

### 2.4. Conventional Group

Reconstruction in the conventional group was performed using the standard “freehand” technique, without virtual planning, relying on the surgeon’s intraoperative judgment and technical skill for bone segmentation and flap adaptation.

Mandibular osteotomies were executed using standard instruments—reciprocating saw or piezoelectric scalpel—and resection margins were defined preoperatively based on imaging findings and confirmed intraoperatively according to the visible and palpable extent of the lesion or tumor. The osteocutaneous free fibula flap was harvested following standard microsurgical techniques. Fibular osteotomies were manually planned and performed intraoperatively, based on direct measurement of the defect with a ruler or using the resected mandibular segment as a template. Segmentation and adjustment were empirically performed until an approximation of the native mandibular contour was achieved, depending largely on the surgeon’s experience.

A standard 2.5-mm titanium reconstruction plate, not patient-specific, was used. This plate was manually bent and adapted to match the shape of the segmented fibula flap and to connect the fibular bone segments with each other and the residual mandible. The average cost was approximately 2500 € (including other components such as screws or drilling devices).

### 2.5. CAD/CAM Group

To initiate virtual surgical planning, the previously described CT scans of the maxillofacial skeleton and both tibioperoneal trunks were obtained for all patients. The CT data were exported in DICOM (Digital Imaging and Communications in Medicine) format and transferred to a CAD/CAM software platform, FreeformPlus v2024.0.87. The regions of interest (mandible, fibula, etc.) were manually segmented and converted into three-dimensional virtual models using proprietary software (IPS CaseDesigner^®^ KLS Martin Group).

Subsequently, a multidisciplinary session was held between the Department of Oral and Maxillofacial Surgery and KLS Martin engineers to define virtual osteotomies, determining resection margins (at least 1 cm in malignant tumors). Virtual planning was performed using the mentioned software, and specific cutting guides were designed for both the mandible and the fibula.

The virtual cutting guides were produced via additive manufacturing using selective laser sintering (SLS) technology with polyamide material. A 3D-printed model of the patient’s reconstructed mandible was also generated. This reconstructed mandible model was used to verify the spatial arrangement of the fibular segments and confirm the positioning of the reconstruction plate.

**Figure 1:**
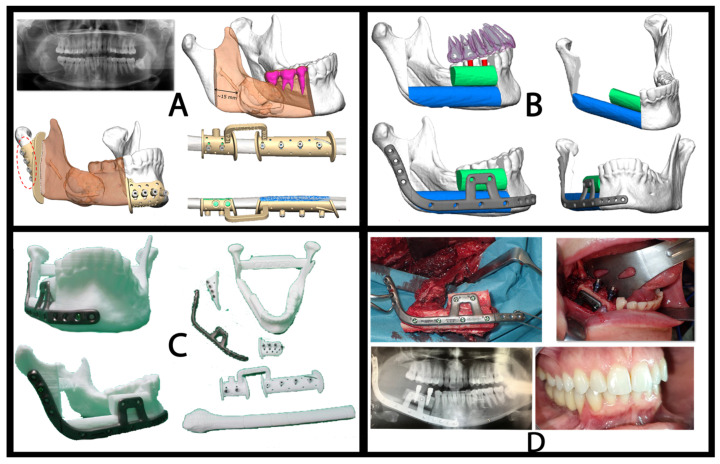
Example of CAD/CAM workflow (KLS Martin) for fibula free flap mandibular reconstruction. (**A**) Preoperative imaging and virtual surgical planning. Preoperative orthopantomography showing the initial mandibular condition, followed by three-dimensional virtual planning of the mandibular resection and the fibular osteotomies. (**B**) Virtual simulation of fibula flap adaptation and reconstruction. Computer-assisted planning of the fibula free flap reconstruction, including segmentation and positioning of fibular bone segments to restore mandibular continuity. The virtual reconstruction is assessed both without and with a customized patient-specific reconstruction plate, visualized from multiple perspectives to evaluate alignment, contour, and spatial accuracy. (**C**) Patient-specific biomodels and surgical devices. Three-dimensional printed biomodels of the mandible, patient-specific cutting guides, and the customized reconstruction plate. These models are used for preoperative validation of the surgical plan, verification of guide fit, and optimization of intraoperative workflow. (**D**) Intraoperative execution and postoperative outcome. Intraoperative shaping and fixation of the osteofasciocutaneous fibula free flap using CAD/CAM guides and a customized reconstruction plate, followed by postoperative orthopantomogram. Final clinical outcome includes dental implant placement and restoration of occlusion consistent with the preoperative virtual planning.

Virtual planning and 3D printing were outsourced (Figure 1). The total process—planning, design, manufacturing, shipping, and delivery—took five business days. The printed models, cutting guides, and reconstruction plate were sterilized using steam sterilization technology in accordance with DIN EN 13060/DIN EN 285 or ANSI AAMI ST79 standards (American National Standard. AAMI: Arlington, VA, United States of America, 2017) [6] and prepared for intraoperative use. The reconstruction was performed using a standard load-bearing 2.5-mm titanium reconstruction plate system with monocortical screw fixation. The average cost of virtual planning and all associated components of free fibula flap reconstruction was approximately 9.000 €.

### 2.6. Statistical Analysis

Continuous variables were expressed as mean ± standard deviation or median (interquartile range), as appropriate, and compared between groups using Student’s *t*-test or the Mann–Whitney *U* test, depending on data distribution. Categorical variables were summarized as frequencies and percentages and compared using the chi-square test or Fisher’s exact test, as appropriate. Normality of continuous variables was assessed using the Shapiro–Wilk test.

Perioperative clinical variables, postoperative outcomes, oncologic margin status, and institutional cost components were compared between CAD/CAM-guided and conventional freehand reconstruction groups. Operative time, hospital and intensive care unit (ICU) length of stay, perioperative blood transfusion requirement, postoperative complications, flap failure, reintervention rate, and margin status were analyzed as outcomes of interest.

To evaluate the independent association between reconstruction technique and perioperative outcomes, multivariate linear regression models were constructed with operative time and hospital length of stay as dependent variables. Reconstruction technique (CAD/CAM-guided versus conventional freehand) was entered as the main independent variable. Covariates were selected a priori based on clinical relevance and included age, perioperative blood transfusion requirement, and margin status. To minimize the risk of model overfitting given the sample size, only a limited number of clinically relevant covariates were included in the final models.

Given the retrospective nature of the study, no a priori sample size calculation was performed. All statistical tests were two-tailed, and statistical significance was set at *p* < 0.05. Statistical analyses were performed using SPSS software (version 31.0.1.0), IBM Corp., Armonk, NY, USA). Temporal trends related to the progressive institutional adoption of CAD/CAM technology were acknowledged as a potential source of bias and were considered in the interpretation of results.

### 2.7. Use of Generative Artificial Intelligence

During the preparation of this manuscript, ChatGPT (OpenAI, GPT-5.2) was used solely for language editing and formatting under the supervision of the authors. No AI tool was employed for data generation, analysis, or interpretation. The authors reviewed and verified all content for accuracy and scientific integrity

## 3. Results

A total of 51 patients undergoing mandibular or maxillary reconstruction of the jaw with an osteocutaneous free fibula flap were included: 25 treated with CAD/CAM-guided reconstruction and 26 with the conventional freehand technique. Group allocation reflected a temporal implementation pattern, with conventional freehand reconstruction performed exclusively between 2017 and 2020, and CAD/CAM-guided reconstruction performed from 2021 onward. Baseline characteristics showed that the CAD/CAM group had a lower mean age compared with the conventional freehand group (57.2 ± 17.4 vs. 64.1 ± 14.1 years; *p* = 0.1306). No significant differences were observed in sex distribution between groups. Baseline demographic and clinical characteristics are summarized in Table 1.

**Figure 2:**
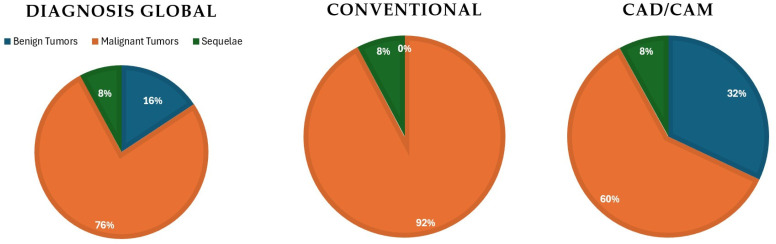
Distribution of primary diagnoses by reconstructive technique. The freehand cohort predominantly included malignant tumors, particularly squamous cell carcinoma, whereas the CAD/CAM-guided group showed a more heterogeneous diagnostic distribution with a higher proportion of benign but anatomically complex lesions. This distribution reflects the temporal implementation of CAD/CAM-guided reconstruction from 2021 onward.

A diagnostic breakdown showed distinct patterns between groups (Figure 2). The conventional freehand cohort predominantly included malignant tumors, with squamous cell carcinoma accounting for most cases (17/26). In contrast, the CAD/CAM-guided group demonstrated a more heterogeneous diagnostic distribution, including a higher proportion of benign but surgically complex lesions such as ameloblastomas (6 cases), as well as soft tissue and bone sarcomas and post-oncologic reconstructions. A diagnostic breakdown showed distinct patterns between groups (Figure 2). The conventional freehand cohort predominantly included malignant tumors, with squamous cell carcinoma accounting for most cases (17/26), whereas the CAD/CAM-guided group showed a more heterogeneous diagnostic distribution, including ameloblastomas, sarcomas, and post-oncologic reconstructions.

To address potential diagnostic imbalance, we emphasize that group allocation followed a temporal institutional implementation of CAD/CAM-guided reconstruction rather than case selection based on pathology. Therefore, diagnostic differences reflect evolving practice patterns rather than preferential assignment of specific diagnoses to a given technique. Subgroup comparison restricted to identical diagnostic categories was not feasible due to sample size limitations.

**Figure 3:**
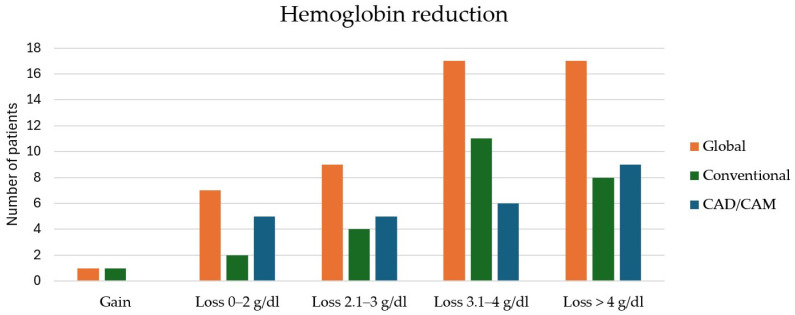
Perioperative blood loss and transfusion requirements. Comparison of immediate postoperative hemoglobin decrease and perioperative blood transfusion rates between CAD/CAM-guided and conventional freehand fibula free flap reconstructions. Postoperative hemoglobin decrease was comparable between groups; however, perioperative blood transfusion was required less frequently in the CAD/CAM-guided cohort.

Tracheostomy was performed in all patients treated with the conventional freehand technique (100%) and in 92% of patients in the CAD/CAM-guided group. The mean immediate postoperative hemoglobin decrease was 3.32 ± 1.46 g/dL in the CAD/CAM group and 3.49 ± 1.53 g/dL in the freehand group. Perioperative blood transfusion was required more frequently in the freehand group (21 vs. 10 patients; *p* = 0.004) (Figure 3).

Perioperative outcomes are summarized in Table 2. CAD/CAM-guided reconstruction was associated with a significantly shorter operative time and a shorter hospital stay in univariate analysis. ICU stay did not differ significantly between groups. Margin status did not differ significantly between groups.

In multivariate linear regression analysis adjusting for age, perioperative blood transfusion requirement, and margin status, CAD/CAM guidance was not independently associated with hospital length of stay. Although the model estimated a mean reduction of 6.5 days in hospital stay for the CAD/CAM group, this association did not reach statistical significance (*p* = 0.288). Perioperative blood transfusion requirement showed the largest effect estimate, with an average increase of 7.5 days in hospital stay; however, this association was also not statistically significant.

Maxillary defects were classified according to the Brown and Shaw classification, which stratifies maxillectomy defects based on vertical extent (Classes I–IV) and horizontal involvement (subclasses a–d), allowing standardized description of defect complexity and reconstructive requirements [7]. Mandibular defects were classified using the Brown classification system, which categorizes segmental mandibulectomy defects into four main classes (I–IV) based on defect location and extent, with the optional suffix “c” indicating condylar involvement [8]. This classification provides a reproducible framework for assessing mandibular defect complexity and guiding reconstructive strategy.

After exclusion of one patient with no bone resection performed, maxillar defect classification was available for 4 cases and mandibular defect classification for 46 cases. Brown class II defects were the most frequent (45.7%), followed by class I (21.7%) and class IV (21.7%), while class III defects accounted for 10.9%. Condylar involvement (“c” subtype) was identified in four mandibular cases (8.7%), occurring in class I (*n* = 2), class II (*n* = 1), and class IV (*n* = 1), with no condylar involvement observed in class III defects.

Maxillary defects were infrequent in this series, accounting for a small subset of the total cohort. According to the Brown and Shaw classification, the maxillectomies included a limited number of advanced defects, reflecting the low prevalence of maxillary resections requiring microvascular reconstruction in our population. Given the small number of cases, maxillary defects were described descriptively without further stratified analysis.

Regarding fibular segmentation, most reconstructions were performed using two fibular segments in a conventional configuration (*n* = 29, 56.9%), followed by single-segment (*n* = 11, 21.6%) and three-segment reconstructions (*n* = 5, 9.8%). A double-barrel fibula configuration was employed in six cases (11.7%). Among these, three reconstructions used two segments, two used three segments, and one used four segments. Double-barrel cases were analyzed as a separate reconstructive subgroup due to their distinct biomechanical and functional characteristics.

Inferential analysis between operative time and individual Brown mandibular defect classes was not performed due to the small sample size within certain categories, which precluded reliable statistical comparison.

No significant association was observed between operative time and the number of fibular segments used for reconstruction (ANOVA *p* = 0.776; Kruskal–Wallis *p* = 0.769). No significant difference in operative time was observed between double-barrel and non-double-barrel fibula reconstructions (*p* = 0.659).

### Cost Analysis

Cost results are reported descriptively as mean estimated institutional costs per patient. Using the institutional cost structure—40 € per operating-room minute, 550 € per day in a standard hospital ward, 1400 € per day in the intensive care unit (ICU), and 200 € per unit of transfused blood—together with guide-related expenses (9000 € for CAD/CAM planning and 2500 € for a standard reconstruction plate and screws), the mean total cost per patient was calculated for each cohort.

For patients treated with CAD/CAM-guided reconstruction, the mean operative time of 542.3 min corresponded to an estimated operating-room cost of 21,692 €. The average ICU stay (2.88 days) generated an additional cost of 4032 €, while the mean postoperative ward stay (19.56 days) contributed 10,758 €. The mean transfusion-related cost was minimal (120 €, corresponding to an average of 0.6 units of transfused blood), reflecting the lower transfusion requirement observed in this group. Including the 9000 € cost of virtual planning and manufacturing, the total estimated cost per CAD/CAM patient was 45,602 €.

**Figure 4:**
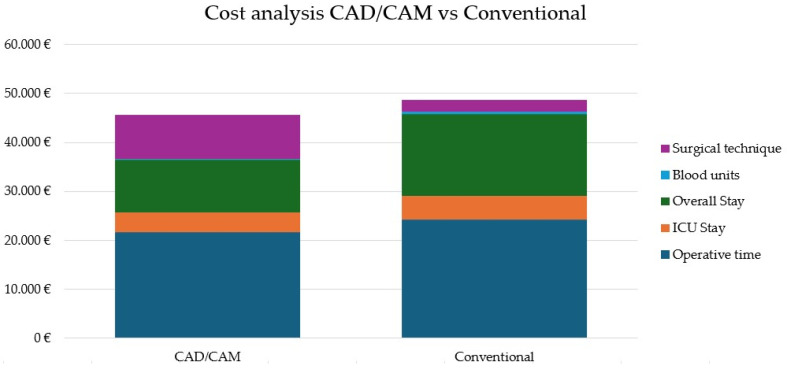
Comparative cost analysis of CAD/CAM-guided and conventional freehand reconstruction. Breakdown of the estimated total institutional cost per patient for jaw reconstruction with a free fibula flap performed using CAD/CAM-guided planning versus the conventional freehand technique. Cost components include operating-room time, intensive care unit stay, standard ward hospitalization, blood transfusions, and reconstruction-related device expenses. Hospital ward stay represented the largest single component of total institutional cost in both cohorts, explaining its role as the principal cost driver in the economic model. Despite higher upfront planning and manufacturing costs, the CAD/CAM-guided cohort showed a lower estimated overall cost per patient, primarily associated with reduced operative time and lower transfusion-related costs.

In the freehand cohort, the mean operative time of 604.9 min resulted in an estimated operating-room cost of 24,196 €. The average ICU stay (3.46 days) and ward stay (30.6 days) represented costs of 4844 € and 16,830 €, respectively. Transfusion-related costs were higher (370 €, corresponding to an average of 1.85 units of transfused blood), consistent with the significantly increased transfusion requirement observed in this group. After adding the 2500 € cost of a standard reconstruction plate, the total estimated cost per freehand patient was 48,740 € (Figure 4).

## 4. Discussion

In this retrospective study comparing CAD/CAM-guided versus conventional freehand fibula free flap reconstruction, several clinically relevant differences in perioperative efficiency and institutional resource utilization were identified. In contrast to earlier reports suggesting comparable operative metrics between techniques, our analysis demonstrated a significantly shorter operative time and a reduction in hospital length of stay in univariate analysis for the CAD/CAM-guided cohort, supporting the concept that virtual surgical planning (VSP) and patient-specific guides can translate into measurable efficiency gains when fully integrated into institutional workflows. Importantly, intensive care unit (ICU) stay, flap failure, and reintervention rates did not differ significantly between groups, findings that are consistent with multiple previously published series [9,10,11,12,13].

The reduction in operative time observed in the CAD/CAM-guided group aligns with previous studies reporting improved intraoperative efficiency through preplanned osteotomies, precise fibular segmentation, and optimized plate adaptation. Preoperative definition of resection margins and fibular cuts minimizes intraoperative trial-and-error, repeated bone handling, and time-consuming manual contouring, all of which are recognized contributors to prolonged operative duration. Powcharoen et al. and Rodríguez-Arias et al. reported reductions in reconstructive time associated with CAD/CAM-assisted workflows, although not all series have demonstrated statistically significant differences [9,13]. Variability across studies likely reflects differences in institutional experience, case complexity, and the phase of technology adoption. In our series, CAD/CAM was progressively implemented over several years, and the observed time savings may reflect increasing familiarity with digital planning, guide positioning, and multidisciplinary coordination rather than the planning modality alone.

Although hospital length of stay was shorter in the CAD/CAM group in univariate analysis, this association did not remain statistically significant after multivariate adjustment, underscoring the multifactorial nature of postoperative recovery. Length of stay is influenced by multiple interrelated factors, including transfusion requirement, patient-related characteristics, and underlying pathology, rather than by planning modality alone. Rather than contradicting the univariate findings, these results suggest that CAD/CAM guidance may contribute indirectly to reduced hospitalization through improvements in perioperative efficiency and reduced transfusion requirements, rather than acting as an independent determinant of recovery.

One of the most robust and clinically relevant findings of the present study was the significantly lower need for perioperative blood transfusion in the CAD/CAM-guided cohort. Despite comparable immediate postoperative hemoglobin decreases, fewer patients required transfusion and the mean number of transfused units was lower in the guided-planning group. This observation suggests that CAD/CAM-assisted reconstruction may reduce variability in intraoperative blood loss, likely by limiting unplanned osteotomies, repeated bone handling, and prolonged plate adaptation. Reduced transfusion requirements are clinically meaningful given their established associations with postoperative morbidity, immunomodulation, and increased healthcare costs. Notably, this finding has received relatively limited emphasis in prior literature, although some authors have suggested that improved efficiency and reduced ischemia times associated with CAD/CAM workflows may mitigate physiological stress during surgery [9,11].

Demographic characteristics were broadly comparable between groups, although patients in the CAD/CAM cohort were younger, a difference that likely reflects temporal practice patterns rather than selection bias. Cardiovascular risk factors and toxic habits were evenly distributed and not statistically different, consistent with previous reports indicating that comorbidity profiles should not drive the choice of reconstructive technique [11,14]. The rate of tracheostomy did not differ substantially between groups, suggesting comparable airway management requirements irrespective of planning modality.

Margin status remains a critical consideration in oncologic reconstruction. Although differences in margin involvement between CAD/CAM-guided and freehand reconstructions did not reach statistical significance, a higher proportion of negative margins was observed in the CAD/CAM cohort. This finding is consistent with Goetze et al., who demonstrated that CAD/CAM-guided resections do not compromise oncologic safety and that bony margins remain reliable when cutting guides are used [10]. However, as emphasized in prior studies, soft-tissue margin involvement rather than bony margins remains the principal predictor of recurrence, highlighting the need for careful intraoperative judgment in tumors with uncertain soft-tissue extension.

The heterogeneity of diagnostic indications between groups represents an important factor in interpreting our findings. While malignant tumors, particularly squamous cell carcinoma, predominated in the freehand cohort, the CAD/CAM group included a higher proportion of benign but anatomically complex lesions such as ameloblastomas. This difference likely reflects the temporal implementation of CAD/CAM-guided reconstruction and evolving institutional practice patterns rather than technique-based case selection. Diagnostic heterogeneity may influence operative duration, transfusion requirements, and airway management, as malignant cases typically require wider resections and more extensive soft-tissue and neck procedures [15,16]. Differences in underlying pathology may also have influenced transfusion requirements, as oncologic resections typically involve more extensive soft-tissue dissection and higher bleeding risk. This potential confounding effect is acknowledged as a limitation.

A direct comparison restricted to identical diagnostic subgroups was not statistically feasible due to sample size constraints. Nevertheless, allocation followed a temporal institutional adoption of CAD/CAM rather than diagnostic case selection, and diagnostic heterogeneity is acknowledged as a limitation. Future multicenter studies with larger cohorts will be required to perform diagnosis-matched comparisons.

Unlike many previous studies focusing primarily on geometric accuracy or reconstructive precision, the present investigation emphasizes perioperative efficiency and real-world institutional resource utilization within a before–after implementation framework. In particular, the observed reduction in blood transfusion requirements represents a clinically relevant and relatively underreported benefit of CAD/CAM-guided reconstruction, with direct implications for patient safety and healthcare expenditure.

From a functional perspective, long-term mandibular function, patient-reported outcomes, and prosthodontic rehabilitation were not evaluated in this study. Previous investigations have reported no clear functional superiority of CAD/CAM over conventional reconstruction with respect to mandibular mobility, with outcomes more strongly influenced by defect extent and adjuvant radiotherapy [11]. Nevertheless, accurate three-dimensional reconstruction may facilitate downstream treatment phases, particularly dental rehabilitation. Salinero et al. demonstrated in a systematic review and meta-analysis that CAD/CAM-assisted mandibular reconstruction is associated with a higher likelihood of dental implant placement compared with conventional techniques [17]. Such downstream benefits were not captured in our dataset but remain clinically relevant.

The economic analysis constitutes a key contribution of this study. Despite higher upfront costs related to virtual planning and patient-specific devices, the total estimated institutional cost per patient was lower in the CAD/CAM-guided cohort, driven primarily by reduced operative time, shorter ward stay, and lower transfusion-related costs. Importantly, this analysis was based on real institutional cost data rather than theoretical estimates, providing a pragmatic assessment of economic impact. Previous studies have reported mixed economic results, with some demonstrating potential savings through efficiency gains [9,13] and others emphasizing the additional cost of virtual planning [11]. Our findings suggest that when institutional workflows are optimized, CAD/CAM-guided reconstruction can be economically advantageous.

The learning curve associated with CAD/CAM implementation likely influenced our results. VSP requires coordination between surgeons, engineers, and manufacturing providers, and efficiency tends to improve as institutional experience accumulates [18,19,20]. Awad et al. described a short learning curve for VSP skill acquisition, with progressive reductions in operative time as workflows matured [18]. Similarly, Vranckx et al. emphasized the importance of institutional adaptation when implementing CAD/CAM protocols [19]. Early-phase adoption may attenuate efficiency gains, whereas mature implementation is more likely to reveal the benefits described in later series and meta-analyses [9,21,22].

This study has limitations inherent to its retrospective, single-center, non-randomized before–after design, which precludes causal inference and introduces potential temporal bias. The sample size limits statistical power for infrequent outcomes such as flap failure and reintervention. Diagnostic heterogeneity and evolving institutional expertise may have influenced perioperative outcomes, and the descriptive economic analysis may not be generalizable to other healthcare systems. Functional outcomes, patient-reported quality of life, and long-term dental rehabilitation were not assessed, limiting conclusions regarding long-term recovery. Although a higher number of fibular segments may intuitively reflect increased reconstructive complexity, no significant association with operative time was observed in the present cohort. This suggests that operative duration may be more strongly influenced by factors such as surgical planning, workflow optimization, and team experience rather than fibular segmentation alone or type of osteotomy. Allocation to CAD/CAM-guided or freehand reconstruction followed a temporal institutional implementation rather than selection based on defect type or complexity. Future prospective studies should incorporate standardized defect classification and segmentation data to allow complexity-matched comparisons between reconstructive techniques.

Despite these limitations, the present study adds meaningful evidence that CAD/CAM-guided fibula free flap reconstruction offers reliable oncologic safety and selected perioperative advantages, particularly reduced operative time and transfusion requirements, while also demonstrating potential institutional cost savings.

Future multicenter prospective studies incorporating functional outcomes, patient-reported measures, and cost-effectiveness analyses will be essential to further define high-value indications for CAD/CAM-guided reconstruction [23,24,25].

## 5. Conclusions

In this retrospective comparative study, CAD/CAM-guided fibula free flap reconstruction was associated with improved perioperative efficiency and more favorable institutional resource utilization compared with the conventional freehand technique. Guided reconstruction resulted in significantly shorter operative time and lower perioperative blood transfusion requirements, while maintaining comparable rates of flap survival, reintervention, and oncologic margin status. Hospital length of stay was shorter in univariate analysis but not after multivariate adjustment, reflecting the multifactorial determinants of postoperative recovery. Despite higher upfront planning and device-related costs, the total estimated institutional cost per patient was lower in the CAD/CAM cohort. Overall, these findings provide real-world evidence that CAD/CAM-guided fibula free flap reconstruction offers selected perioperative and economic advantages while maintaining comparable oncologic outcomes, supporting its value within optimized institutional workflows.

## Figures and Tables

**Table 1:** Baseline demographic and clinical characteristics of the study population. Distribution of age, sex, cardiovascular risk factors, tobacco and alcohol exposure, and year of surgery among patients undergoing bone reconstruction with a free fibula flap using CAD/CAM-guided planning or the conventional freehand technique. Age is presented both as categorical distribution and as mean ± standard deviation.

		Study Population (*n* = 51) (25 CAD/CAM vs. 26 Conventional)					
Variables	Categories	Frequency	(%)	CAD/CAM	(%)	Conventional	(%)
	<40	9	17.6	7	28	2	7.7
Age (years)	40–60	11	21.6	5	20	6	23.1
	>60	31	60.8	13	52	18	69.2
Age (years)		60.7 ± 16		57.2 ± 17.4		64.1 ± 14.1	
Sex	Male	26	50.9	14	56	12	46.2
	Female	25	49.1	11	44	14	53.8
Tobacco	Absent	29	56.9	17	68	12	46.2
	Present	22	43.1	8	32	14	53.8
Alcohol	Absent	41	80.4	23	92	18	69.2
	Present	10	19.6	2	8	8	30.8
Cardiovascular risk factors	Absent Present	24 27	47.1 52.9	13 12	52 48	11 15	42.3 57.7
Date of surgery	2023–2024 2021–2022 2019–2020 2017–2018	15 10 12 14	29.4 19.6 23.5 27.4	15 10 0 0	60 40 0 0	0 0 12 14	0 0 46.15 53.8

**Table 2:** Comparison of perioperative outcomes between CAD/CAM-guided and conventional freehand reconstruction. Operative time, hospital and intensive care unit (ICU) length of stay, perioperative hemoglobin decrease, blood transfusion requirements, and oncologic margin status in patients undergoing fibula free flap reconstruction of the jaw using CAD/CAM-guided planning versus the conventional freehand technique. Values are presented as mean ± standard deviation or percentage, as appropriate.

		Study Population (*N* = 51)	
VARIABLES	CAD/CAM (25)	Conventional (26)	*p*-Value
Age	57.2 ± 17.4 years	64.1 ± 14.1	0.1306
Hemoglobin decrease	3.32 ± 1.46 g/dL	3.49 ± 1.53	0.689
Blood transfusion need	40%	80.8%	0.004
Operative time	542.3 ± 65.8 min	604.9 ± 79.5	0.0036
Hospital stay	19.56 ± 7.2 days	30.6 ± 26.2	0.0467
ICU stay	2.9 ± 2.9 days	3.5 ± 2.5	0.454
Affected margins	24%	38.5%	0.169

## Data Availability

The data presented in this study are available on request from the corresponding author.
